# Robust colonoscopy polyp segmentation using dynamic-Nu T-Loss with multi-scale and uncertainty-aware adaptation

**DOI:** 10.3389/fmed.2025.1657123

**Published:** 2026-01-12

**Authors:** Alireza Norouziazad, Mahan Najafpour Ghazvini Fardshad, Fatemeh Esmaeildoost, Mehrdad Najafpour Ghazvini Fardshad, Razieh Salahandish

**Affiliations:** 1 Laboratory of Advanced Biotechnologies for Health Assessments (LAB-HA), Lassonde School of Engineering, York University, Toronto, ON, Canada; 2 Department of Electrical Engineering and Computer Science (EECS), Lassonde School of Engineering, York University, Toronto, ON, Canada; 3 Department of Mechanical Engineering, Lassonde School of Engineering, York University, Toronto, ON, Canada

**Keywords:** colonoscopy image analysis, deep learning in medical imaging, dynamic-Nu T-Loss, multi-scale aggregation, polyp segmentation, robust loss function

## Abstract

Accurate segmentation of polyps in colonoscopy images is essential for early colorectal cancer detection; however, it remains a challenging task due to reflections, occlusions, motion artifacts, inter- and intra-polyp appearance variability, and the presence of noisy or inconsistent ground-truth annotations. In this work, we introduce dynamic-Nu T-Loss (DNA-TLoss), a robust, adaptive loss function based on the heavy-tailed Student’s 𝑡-distribution that incorporates three novel extensions: (1) a per-image learnable degrees-of-freedom parameter *ν*, predicted by a lightweight NuPredictor network to dynamically adjust robustness to outliers; (2) per-pixel precision weights *λ* for spatially adaptive error sensitivity; and (3) a multi-scale aggregation scheme that computes and combines loss at multiple spatial resolutions to capture both coarse and fine details. Integrated into a U-Net with a ResNet-34 encoder, DNA-TLoss was evaluated on five public benchmarks: CVC-300, CVC-ClinicDB, ETIS-LaribPolypDB, Kvasir, and CVC-ColonDB. Our method achieves the lowest Hausdorff distance across all datasets, with an average reduction of 14.6% compared to T-Loss; notably, on CVC-300, it yields a significant decrease of 45.96%. It also obtains the lowest false discovery rate on all five datasets, improving over T-Loss by up to 38.7% on CVC-300 and 24.5% on Kvasir. Furthermore, DNA-TLoss provided best-in-class calibration, achieving expected calibration error as low as 0.44% on CVC-300 and outperforming all other baselines on four out of five datasets. These results highlight the promise of joint global and local uncertainty adaptation, coupled with multi-scale optimization, for advancing trustworthy, real-time computer-aided polyp detection in colonoscopy.

## 1 Introduction

Colonoscopy is widely used for the early detection and prevention of colorectal cancer. It enables direct visualization of the colon and rectum, allowing early identification of lesions or polyps before they become malignant (1). In the context of medical imaging analysis, accurate medical image segmentation plays an important part in the diagnosis and determining which treatment should be done. Segmentation aids in identifying anatomical structures and localizing abnormalities or lesions accurately (2). This involves the segmentation of polyps, which are clinically significant structures; incomplete segmentation may lead to false negatives, potentially resulting in missed or delayed diagnoses (3). Routine colonoscopy has been reported to miss 17–28% of polyps, especially small and flat lesions, during conventional colonoscopy (4). When an AI-assisted colonoscopy is performed prior to the traditional examination, the sensitivity of the subsequent procedure decreases by 4.76%, not due to diminished effectiveness, but because the AI system has already identified a significant number of lesions (5). Annotation detail significantly impacts performance; models trained on precise outline masks achieve 99.6% sensitivity, while those using coarser, 20%, inflated bounding boxes drop to 94.84% (5).

Unlike general medical imaging, segmentation of colonoscopy data presents unique challenges, including intense mucosal reflections from the colonoscopy light source, frequent fluid occlusions obscuring the field of view, motion artifacts from peristalsis or camera movement, and substantial variability in polyp size, shape, and texture (6).

Deep learning models are often trained on noisy or poor-quality annotations due to human errors in labeling, and the limited expert staff who can annotate the data at a high quality (2, 7–9). Label noise is a well-known challenge and can significantly impact model performance, as noise leads to uncertainty during training, which can lead to poor generalization and unsafe predictions (10). The inter-annotator average Dice coefficient found in previous studies (e.g., polyp segmentation and MRI) ranges from 0.72 to 0.88 (11). There are also well-documented instances of low-quality medical imaging, arising from inter-expert discrepancies in annotations, ambiguous cases, and inconsistent policies in the medical field (12–14). To address these challenges, we introduce a deep neural network model including preprocessing, an encoder-decoder architecture, evaluation metrics and a loss function.

Loss functions make a significant impact in training machine learning models, particularly in clinical settings where prediction errors can have serious consequences. In classification tasks, loss functions that are commonly applied include cross-entropy (15), hinge loss (16), and exponential loss (17), while regression tasks often apply squared error (18), absolute error, or robust alternatives, such as Huber loss (19).

In challenging tasks like object detection and semantic segmentation, traditional loss functions in deep learning may be impacted by class imbalance and noisy labels. Different variations of loss functions for noisy labels are shown to improve spatial accuracy and feature separability, such as focal loss (20), IoU-based loss (21), standard contrastive loss (11), and ArcFace loss (22). While these approaches have demonstrated improved spatial precision, they remain susceptible to annotation errors commonly found in medical data (19).

Successful robust loss functions in other domains cannot help with colonoscopy tasks because they assume i.i.d. noise, whereas colonoscopy errors are structured. The rich measured noise that comes from colonoscopy data is, however, fundamentally problematic: the extent of systematic errors that occur in colonoscopy is not amenable to robust loss functions in a standard way (12). Robust loss functions help ensure reliable model performance in clinical settings (23). They handle noisy annotations and reduce the impact of outliers. This leads to greater precision and reliability in predictions. As a result, diagnoses are safer, and clinical benefits increase (24).

Some loss functions, such as those proposed in Patrini et al. (25), incorporate a noise transition matrix to adjust how the loss is applied. Subsequent work (26) further advanced this approach by modeling the transition matrix more accurately, thereby reducing the impact of label noise on the effective loss. Robust loss functions, like Generalized Cross Entropy (27) or Symmetric Cross Entropy (28), do not explicitly model label noise directly but have demonstrated strong performance in noisy settings. Other strategies for enhancing robustness include outlier-aware loss functions (29) and normalized loss functions (30), which aim to mitigate the impact of mislabeled data. Both Zhang and Sabuncu (27) and Wang et al. (28) also contribute to this effort by addressing robustness from a theoretical and empirical perspective. The only difference between these two approaches is whether noise is modeled directly in the loss functions. Anticipating noise is indeed a different approach to tackling the problems of noise, using the design of the loss function.

Here, we build upon the Student’s-𝑡-based T-loss (31), a robust loss function that models label ambiguity using the heavy-tailed Student’s 𝑡-distribution. We extend this framework by proposing a new variant, dynamic-Nu T-Loss (DNA-TLoss), which introduces additional adaptability and robustness to varying data conditions. A key innovation of DNA-TLoss is the introduction of a learnable degree of freedom parameter, which adjusts dynamically to image-level label ambiguity during training. This dynamic modeling allows for improved classification behavior on ambiguous annotations.

This paper is organized as follows: Section 2 introduces the datasets used for evaluation, including Kvasir (32), CVC-ClinicDB (33), CVC-ColonDB (34), ETIS-LaribPolypDB (35), and CVC-300 (36). It also details the network architecture, defines the problem setting, outlines the motivation for the proposed DNA-TLoss, and provides its mathematical formulation. Section 3 presents the experimental results, including quantitative performance across multiple polyp segmentation benchmarks using metrics such as Dice, Intersection-over-Union (IoU), false discovery rate (FDR), Hausdorff distance (HD), average symmetric surface distance (ASSD), expected calibration error (ECE), and mutual information (MI). Finally, Section 4 concludes the study by summarizing our main contributions and discussing the broader implications of our findings for robust medical image segmentation.

## 2 Methods

### 2.1 Dataset and preprocessing

We evaluated our method on multiple publicly available colonoscopy polyp segmentation datasets: Kvasir, CVC-ClinicDB, CVC-ColonDB, ETIS-LaribPolypDB, and CVC-300. For training, we used a combined set of 900 randomly selected images from Kvasir and 550 training images from CVC-ClinicDB. Evaluation was performed across all five datasets to assess generalization performance.

CVC-ClinicDB includes 612 high-resolution images; 62 of these were reserved for testing. CVC-ColonDB contains 380 earlier-generation colonoscopy frames, while ETIS-LaribPolypDB comprises 196 challenging test samples with diverse imaging conditions. Kvasir consists of 1,000 annotated frames extracted from real-world colonoscopy videos, showcasing polyps of varying size and morphology. CVC-300 consists of 300 colonoscopy video frames that do not overlap with ClinicDB.

All images were resized to a uniform resolution of 512 × 608 pixels, selected to balance detail preservation with computational efficiency while closely matching the native dimensions of datasets, minimizing interpolation artifacts. To preserve the aspect ratio, zero-padding was applied where needed. During training, we applied comprehensive data augmentation using the Albumentations library to simulate common clinical noise and intra-patient variability. This included horizontal and vertical flipping (*p* = 0.5), elastic deformation (alpha = 1.0, sigma = 50.0, *p* = 0.5), grid distortion (num_steps = 5, distort_limit = 0.3, *p* = 0.5), random adjustments to brightness and contrast within a range of ±20% (*p* = 0.5), and the addition of Gaussian noise with a variance limit of (10.0, 50.0) (*p* = 0.5). After augmentation, each image was normalized using the dataset’s channel-wise mean and standard deviation. These preprocessing steps aimed to improve the model’s robustness to clinical variations in polyp appearance and imaging conditions.

### 2.2 Network architecture

**Figure 1:**
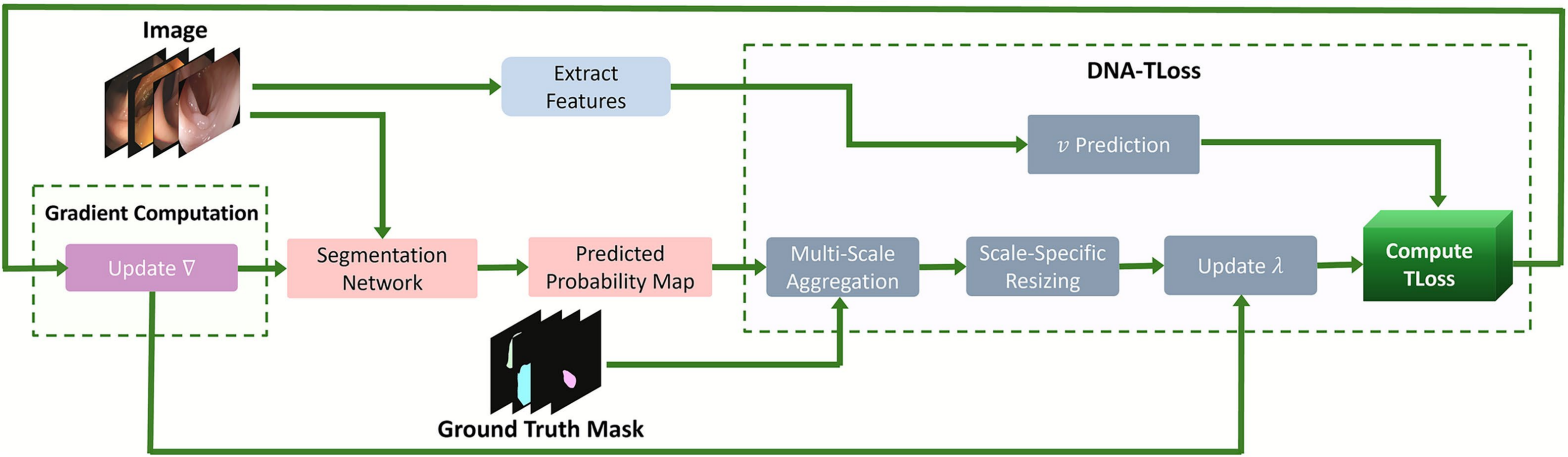
Overview of the DNA-TLoss framework. The model integrates a segmentation network with a NuPredictor module for per-image robustness estimation and per-pixel *λ* maps for adaptive precision. Multi-scale loss aggregation enables improved learning under label noise and structural variability.

The segmentation model employed in this study was a standard U-Net (37) with a pretrained ResNet-34 (38) encoder. The network consists of a contracting path (encoder) to capture contextual features and a symmetric expanding path (decoder) to enable precise spatial localization (39). The input to the model comprises RGB colonoscopy images, and the model produced per-pixel logits for two classes: background and polyp. We converted these logits to a single foreground probability map $p\in {\left[0,1\right]}^{H\times W}$ by applying a sigmoid function for single-channel output, or a Softmax function for two-channel outputs, followed by the polyp class channel. During training, raw logits were directly used for loss computation. At inference time, the resulting probability map was thresholded at 0.5 to produce a binary segmentation mask. The segmentation network was trained using the standard Adam optimizer with a polynomial learning-rate schedule (40). The network outputs a single-channel logit map, which is passed through a sigmoid activation during inference. For training, raw logits were used in the loss computation. The decoder employs skip connections from the encoder to preserve fine spatial information. The overall architecture and data flow of the proposed DNA-TLoss framework are illustrated in Figure 1, highlighting the integration of the NuPredictor module, per-pixel precision adaptation, and multi-scale loss computation within the segmentation pipeline. The segmentation network generates a probability map supervised by the ground-truth mask. Simultaneously, features extracted from the input are passed through a lightweight NuPredictor that estimates a per-image degrees of freedom parameter (*𝜈*), which controls the global robustness of the loss function. The predicted probability map is then resized to multiple scales and aggregated in a multi-scale processing block, where each scale has its own spatially learnable precision map *𝜆*. These *λ* maps adjust local sensitivity to errors by weighting pixel-wise residuals. The DNA-TLoss combines the adaptive *𝜈* and resolution-specific *𝜆* values to compute the final loss. All components, including segmentation parameters, *𝜈*-predictor, and *𝜆*-maps, are jointly optimized via backpropagation, as indicated by the green flow of gradients.

We deliberately adopted a standard U-Net architecture with a ResNet-34 encoder for two key reasons. First, our primary goal was to evaluate the impact of the proposed DNA-TLoss function, rather than to optimize or innovate on the backbone architecture. Using a widely adopted, canonical architecture ensures that improvements can be attributed to the loss function itself, rather than architectural variations. This setup aligns with many prior studies in medical image segmentation, facilitating more direct and meaningful comparisons with existing methods. Second, ResNet-34 offers a balanced trade-off between feature representation capacity and computational efficiency. It is sufficiently deep to extract expressive features while remaining lightweight enough to support real-time inference, which is important in clinical applications such as colonoscopy (38).

### 2.3 Multi-scale dynamic-Nu T-Loss

The core contribution of this work is the introduction of a novel robust loss function, DNA-TLoss, which extends the Student’s-𝑡 negative log-likelihood (T-Loss NLL) (31) to the context of multi-scale semantic segmentation, incorporating image-wise and spatially adaptive robustness mechanisms. This formulation enables the loss to dynamically adjust to both global (image-level) and local (pixel-level) sources of uncertainty, enhancing the model’s resilience to label noise and annotation ambiguity. We briefly review the Student’s-𝑡 formulation that underpins our proposed loss function:

#### 2.3.1 Problem setting

Given an input image $x\in {\mathbb{R}}^{3\times H\times W}$, a segmentation model f(x) predicts a probability map $p\in {\left[0,1\right]}^{1\times H\times W}$, which is compared against a binary ground-truth mask $y\in {\left\{0,1\right\}}^{1\times H\times W}$. The training objective is to minimize a loss function $ℒ(\hat{y},y)$ that encourages pixel-wise agreement between predictions and labels, even in the presence of noise or ambiguous boundaries (41, 42).

Given an input image $x\in {\mathbb{R}}^{3\times H\times W}$, a segmentation model f is the mean, and Σis the covariance matrix. Let δ=y−μ; then $\|\delta^{2}\|\;=\sum_{i}{\left(y_{i}-\mu_{i}\right)}^{2}$corresponds to the mean squared error.

#### 2.3.2 Multi-scale dynamic-Nu

In our DNA-TLoss, we introduce three key extensions to this basic T-Loss:

*Adaptive 𝜈*. Rather than using a fixed degrees-of-freedom parameter, our model predicts a single scalar *𝜈* for each input sample. This global adaptation allows the loss to modulate its robustness based on overall image characteristics: low ν produces heavy-tailed distributions (robust to outliers), while high ν yields Gaussian-like behavior (sensitive to errors). The NuPredictor receives features extracted via three convolutional layers (kernel size = 3 × 3, stride = 1, padding = 1) with ReLU activations. These features are pooled via adaptive average pooling and fed into a two-layer MLP (hidden size = 32, Softplus output) to predict a per-image *ν*. These layers extract high-level features from the input image, which are then passed through adaptive average pooling to yield a fixed-length feature vector per image. This vector is fed into a two-layer MLP (NuPredictor) with a hidden dimension of 32 and a Softplus output activation to ensure positivity. The hidden dimension of 32 was chosen to balance representational capacity and computational cost, large enough to model relevant variations, yet compact enough to avoid overfitting. We use Softplus to ensure positive but non-zero *ν* values, as extremely small *ν* leads to instability. The predicted *𝜈* thus varies across samples, enabling the loss function to modulate robustness based on image content, such as polyp size or image complexity. Importantly, while our architecture processes each image through the NuPredictor during training, empirical analysis reveals that the predicted *ν* values converge to a highly consistent dataset-specific optimum. After training completion, this optimized *ν* value becomes effectively fixed for the entire dataset during inference.

*Per-pixel precision map*. We introduce a learnable precision (inverse-variance) parameter $\lambda_{i}$ for each pixel. Equivalently, we define $\Lambda =\mathrm{diag}(e^{\lambda_{1}},,\ldots ,,e^{\lambda_{D}})$ so that $\Sigma^{-1}=\Lambda$. These parameters are initialized to zero, corresponding to unit precision before training. During optimization, $\lambda_{s,i}>0$ increases the loss penalty for errors at pixel i, while $\lambda_{s,i}<0$ reduces it, allowing the model to learn spatially adaptive error sensitivity. These λ parameters are trained alongside the network. When λ=0, the precision is 1 (unit variance). The log determinant term becomes$-\frac{1}{2}\sum_{i}\lambda_{i}$, and the quadratic term becomes $\sum_{i}\delta_{i}^{2}e^{\lambda_{i}}$. In practice, we add a small constant inside exp/log for stability (omitted below for clarity).

*Multi-scale aggregation*. To effectively capture both coarse and fine details, the proposed loss is computed at multiple spatial resolutions. Specifically, let the original prediction and ground-truth mask be of size (H,W). For each scale s∈1.0,0.5,0.25,with associated weight $w_{s}\in \{1.0,0.5,0.3\}$), we resize the predicted probability map p and ground-truth mask y to $\left(h_{s},w_{s})=(\mathrm{sH},\mathrm{sW}\right)$. Bilinear interpolation is used for resizing the probability map p, while nearest-neighbor interpolation is applied to the ground-truth mask y to preserve categorical labels. At each resolution *s*, we maintain a separate *λ*-parameter matrix $\Lambda_{s}\in R^{h_{s}\times w_{s}}$, as illustrated in Figure 1. This allows each scale to have its own spatial precision map.

The specific scale factors (1.0, 0.5, 0.25) and weights (1.0, 0.5, 0.3) were determined through empirical evaluation on polyp segmentation datasets. We tested various configurations and found this combination optimal for capturing both fine polyp details and contextual information while maintaining computational efficiency suitable for real-time clinical applications.

Combining these components, for one scaled output, let $D_{s}=h_{s}w_{s}$ denote the number of pixels at scale *s,* $\delta_{i}=p_{s,i}-y_{s,i}$ represent the pixel-wise residual, and $\lambda_{s,i}$ denote the corresponding log-precision value. The scale-specific loss is then defined as shown in Equation 3:


(3) $$\begin{array}{l} L_{s}=-\log\Gamma (\frac{\nu +D_{s}}{2})+\log\Gamma (\frac{\nu }{2})-\frac{1}{2}\sum_{i=1}^{D_{s}}\lambda_{s,i}\\ +\frac{D_{s}}{2}\log(\pi )+\frac{D_{s}}{2}\log\;(\nu )+\frac{\nu +D_{s}}{2}\log(1+\frac{\sum_{i=1}^{D_{s}}\delta_{i}^{2}e^{\lambda_{s,i}}}{\nu }) \end{array}$$

This matches the Student’s-𝑡 NLL with $\Sigma^{-1}=\mathrm{diag}(e^{\lambda_{s,i}})$, except for a small constant $\left(\epsilon =10^{-6}\right)$ added inside the logarithmic and exponential terms when implementing the loss. Finally, the total loss is a weighted sum over scales, as defined in Equation 4:


(4) $$L_{\text{Total}}=\sum_{s\in \{1,0,0.5,0.25\}}w_{s}L_{s}$$

All loss parameters (ν and $\lambda_{s,i}$) are updated by backpropagation along with the network weights.

The feature extractor and *ν*-predictor network, as well as the λ parameters, are trained jointly with the segmentation model. In summary, our dynamic-Nu T-Loss automatically learns a per-image scale parameter *ν* and spatial precision weights λ, while aggregating loss at multiple scales.

## 3 Experiments

### 3.1 Implementation details

All experiments are conducted using the PyTorch framework (v1.X) on an NVIDIA A100 GPU. Both the training and evaluation pipelines utilized standard PyTorch modules and custom components for the proposed loss function. The UNet architecture with a ResNet-34 encoder pre-trained on ImageNet (43) was used as the base segmentation model.

The model was trained using the Adam optimizer with an initial learning rate of 0.001, weight decay of 1e−4, and no warm-up schedule (40). A polynomial decay strategy was applied to reduce the learning rate over time, defined as $\eta_{t}=\eta_{0}{\left(1-\frac{t}{T}\right)}^{p}$, where $\eta_{0}$ is the initial learning rate, tis the current training step, *T* is the total number of training steps, and p=0.9 is the polynomial power.

The model was trained for 300 epochs with a batch size of 16. The batch size was chosen based on GPU memory limits while maintaining stable gradient estimates, and 300 epochs were sufficient to ensure convergence without overfitting. All models were trained on pre-separated training and validation splits without cross-validation or ensemble methods. Cross-validation and ensembling were omitted to maintain computational feasibility and isolate the impact of the proposed loss function. Final probability maps from the sigmoid output were thresholded at 0.5 to obtain binary segmentation masks. Performance was evaluated using the Dice coefficient (Dice, higher values indicate better overlap, Equation 5), intersection-over-union (IoU, higher values show better segmentation accuracy, Equation 6), false discovery rate (FDR, lower values indicate fewer false positives, Equation 7), Hausdorff distance (HD, lower values reflect better boundary alignment, Equation 8), average symmetric surface distance (ASSD, lower values demonstrate superior boundary precision, Equation 9), expected calibration error (ECE, lower values represent better model calibration, Equation 10), and mutual information (MI, higher values reveal stronger statistical dependence between predictions and ground truth, Equation 11). These metrics collectively assess the model’s segmentation accuracy, boundary detection reliability, and predictive consistency.


(5) $$\text{Dice}=\frac{2|P\cap G|}{|P|+|G|}$$

(6) $$\mathrm{IoU}=\frac{|P\cap G|}{|P\cup G|}\;\;$$

(7) $$\mathrm{FDR}=\frac{\mathrm{FP}}{\mathrm{TP}+\mathrm{FP}}$$

(8) $$\mathrm{HD}=\max\{\underset{p\in \partial P}{\sup}\underset{g\in \partial G}{\inf}d(p,g),\underset{g\in \partial G}{\sup}\underset{p\in \partial P}{\inf}d(g,p)\}$$

(9) $$\text{ASSD}=\frac{1}{|\partial P|+|\partial G|}(\sum_{p\in \partial P}d(p,\partial G)+\sum_{g\in \partial G}d(g,\partial P))$$

(10) $$\mathrm{ECE}=\sum_{i=1}^{B}\frac{|b_{i}|}{N}|\mathrm{acc}(b_{i})-\text{conf}(b_{i})|$$

(11) $$\mathrm{MI}=\sum_{p\in \{0,1\}}\sum_{g\in \{0,1\}}p(p,g)\log\frac{p(p,g)}{p(p)p(g)}$$

where *P* and *G* denote predicted and ground truth masks, and ∂P and ∂G refer to their corresponding boundaries. TP (true positives) is the number of correctly predicted foreground pixels, FP (false positives) is the number of pixels incorrectly predicted as foreground, and FN (false negatives) is the number of foreground pixels missed by the prediction. The d(·,·) denotes Euclidean distance. For ECE, we used B=10 confidence bins, where $|b_{i}|$ is bin cardinality, $\mathrm{acc}(b_{i})$ and $\text{conf}(b_{i})$ are bin accuracy/confidence, and p(·) is empirical probability. HD and ASSD were computed using boundary point clouds, while ECE and MI were computed using pixel-wise predicted probabilities and ground truth labels. All metrics, except ECE, were averaged per image; ECE was aggregated across all test pixels.

## 4 Results and discussion

We evaluated our method on five public polyp segmentation benchmarks: CVC-300, CVC-ClinicDB, ETIS-LaribPolypDB, Kvasir, and CVC-ColonDB. Table 1 presents a comprehensive comparison between our approach and established baselines. The results indicate that our loss function demonstrates consistently competitive and balanced performance across a range of evaluation metrics.

**Table 1:** Comparative performance of different loss functions across five polyp segmentation datasets.

Loss	↑Dice	↑IoU	↓HD (px)	↓ASSD (px)	↓ECE	↑MI (bits)	↓FDR
CVC-300							
SCE	0.848217	0.790109	28.913981	10.510178	0.006603	0.113721	0.154723
RCE	0.844896	0.779074	33.330659	8.269100	0.008778	0.111326	0.156832
NGCE	0.835202	0.768612	74.067616	19.030924	0.014102	0.112940	0.191401
MAE	0.856784	0.798917	28.074291	5.972153	0.005961	0.113504	0.134332
GCE	0.817179	0.747890	54.612789	15.587761	0.007113	0.103146	0.138141
T-Loss	0.868975	0.800596	35.330346	9.673056	0.006463	0.114369	0.156743
DNA-TLoss	0.898400	0.835400	19.093800	4.267400	0.004400	0.117500	0.096000
CVC-ClinicDB							
SCE	0.887368	0.836867	41.009847	7.893874	0.014810	0.196821	0.094830
RCE	0.856013	0.805149	33.236326	6.157151	0.014565	0.193452	0.120884
NGCE	0.856032	0.802806	36.197746	9.372071	0.013052	0.191038	0.135489
MAE	0.841897	0.793711	32.923948	7.984917	0.014865	0.194616	0.159195
GCE	0.834790	0.787382	36.604960	15.580337	0.012718	0.194496	0.146852
T-Loss	0.886177	0.829220	32.845973	4.835783	0.015707	0.194178	0.094942
DNA-TLoss	0.887835	0.837800	32.823938	4.389348	0.014340	0.194368	0.094253
ETIS-LaribPolypDB							
SCE	0.604502	0.544699	72.692427	28.100090	0.020264	0.101422	0.388739
RCE	0.568277	0.508063	66.151198	22.628657	0.021211	0.094467	0.405009
NGCE	0.537134	0.485925	73.129485	26.418164	0.024467	0.096171	0.463270
MAE	0.510662	0.471646	53.384386	17.451355	0.020455	0.092621	0.487579
GCE	0.535440	0.483640	98.198752	35.353028	0.018918	0.099151	0.442902
T-Loss	0.644637	0.589085	60.029561	25.775716	0.015751	0.109547	0.361469
DNA-TLoss	0.686004	0.622018	50.7914	16.956776	0.014737	0.109671	0.300559
Kvasir							
SCE	0.858363	0.796340	103.616281	18.530834	0.031915	0.264500	0.113847
RCE	0.884590	0.820629	86.342219	12.989334	0.034644	0.269596	0.080661
NGCE	0.874433	0.813873	93.128531	15.236074	0.031581	0.270470	0.093602
MAE	0.853535	0.792440	78.627346	13.287102	0.039339	0.255448	0.094391
GCE	0.858803	0.792654	91.766528	15.297092	0.033541	0.258845	0.098441
T-Loss	0.887325	0.828422	79.984891	12.678060	0.031429	0.270676	0.067762
DNA-TLoss	0.895609	0.835888	73.9504	11.546408	0.030089	0.269198	0.051244
CVC-ColonDB							
SCE	0.694260	0.625050	63.295047	20.088943	0.034721	0.119224	0.223130
RCE	0.642991	0.571606	76.728854	21.054912	0.044300	0.105071	0.293262
NGCE	0.685480	0.615151	81.613077	24.049365	0.038985	0.120934	0.261452
MAE	0.645711	0.578955	68.097889	21.664987	0.041214	0.110510	0.256612
GCE	0.685300	0.608704	83.975428	26.103571	0.036677	0.116719	0.224401
T-Loss	0.701948	0.633327	61.274800	19.706012	0.034732	0.123850	0.261600
DNA-TLoss	0.747400	0.668200	60.072900	19.152300	0.031800	0.124600	0.203600

Metrics include: dice coefficient, intersection-over-union (IoU), Hausdorff distance (HD) (in pixels), average symmetric surface distance (ASSD) (in pixels), expected calibration error (ECE), mutual information (MI) (in bits), and false discovery rate (FDR). Distance metrics (HD, ASSD) report pixel distances, while MI measures information gain in bits.

On CVC-300, our proposed loss function outperformed all baseline methods with a Dice of 89.84% (vs. 86.90% for T-Loss) and IoU of 83.54% (vs. 80.06%), delivering a clear boost in overall overlap. In terms of boundary precision, the HD decreased to 19.09 pixels (a 46% relative reduction from 35.33 pixels) and ASSD to 4.27 pixels (vs. 9.67 pixels), reflecting tighter contour adherence. Moreover, ECE dropped to 0.44%, and mutual information increased to 0.1175 bits, reflecting enhanced calibration and confidence reliability. The method also maintained an FDR of 9.60%, highlighting improved suppression of spurious segmentations.

On the high-quality CVC-ClinicDB images, our loss secured Dice of 88.78% and IoU of 83.78%, marginally ahead of T-Loss by 0.17 and 0.86%, respectively. Its boundary delineation is among the best, with an HD of 32.82 pixels (vs. 32.85 pixels) and an ASSD of 4.39 pixels, demonstrating consistency even in well-contrasted scenes. The ECE of 1.43% matched top-performing losses, ensuring trustworthy probability outputs. Notably, the FDR of 9.43% was the lowest across all methods, underscoring its precise suppression of false positives in varied lighting and texture conditions.

ETIS-LaribPolypDB presents a significant challenge due to its small, low-contrast polyps. Despite this, the proposed method demonstrated improved performance across multiple metrics. It achieved a Dice score of 68.60% (vs. 64.46% for T-Loss) and IoU of 62.20% (vs. 58.91%), marking *a* > 4% absolute improvement in overlap. Boundary metrics also improved, with the HD reduced to 50.79 pixels (vs. 60.03 pixels) and the ASSD to 16.96 pixels (vs. 25.78 pixels), which helps capture fine, faint edges. With ECE = 1.47%, our model maintained more reliable confidence estimates, while the reduced FDR of 30.06% indicates an enhanced robustness to noise and imaging artefacts relative to baseline methods.

On the diverse Kvasir collection, our loss function produced a Dice of 89.56% and IoU of 83.59%, outperforming T-Loss by ∼0.8% in both metrics. It refined boundary localization to HD = 73.95 pixels (vs. 79.98 pixels) and ASSD = 11.55 pixels (vs. 12.68 pixels), crucial for accurately segmenting varied polyp shapes and sizes. While its ECE of 3.01% paralleled the best methods, the standout achievement was an FDR of only 5.12%, the lowest among all losses, demonstrating comparable precision in suppressing false positives across a heterogeneous dataset.

Even on CVC-ColonDB’s noisy, artifact-prone frames, our loss achieved the highest Dice of 74.74% (vs. 70.19% for T-Loss) and IoU of 66.82% (vs. 63.33%). It delivered the smallest HD of 60.07 pixels and the lowest FDR of 20.36%, reflecting robust detection under adverse conditions. Although ASSD was 19.15 pixels (slightly above T-Loss’s 19.71 pixels), the overall gains in overlap, boundary error, and calibration (ECE = 3.18%) confirm its resilience and reliability for challenging real-world colonoscopy data.

**Figure 2:**
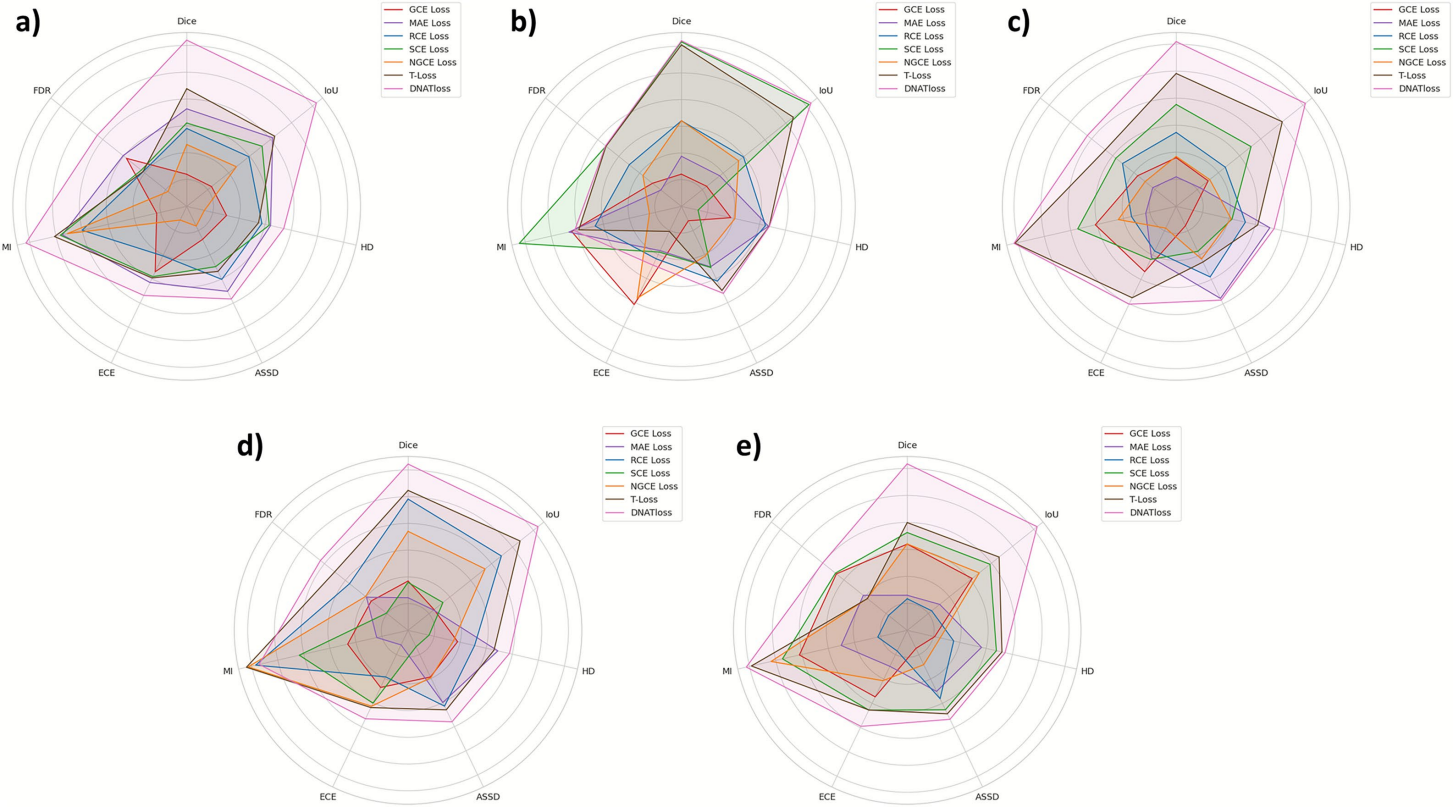
Normalized performance radar charts for different loss functions on five polyp segmentation datasets, including **(a)** CVC-300, **(b)** CVC-ClinicDB, **(c)** ETIS-LaribPolypDB, **(d)** Kvasir, and **(e)** CVC-ColonDB. Each axis represents a performance metric normalized to the [0,1] range, where higher values indicate better performance. Metrics where higher values indicate better performance (Dice, IoU, MI) are plotted directly, while metrics where lower values are preferred (FDR, HD, ASSD, ECE) are inverted during normalization. This normalization scheme ensures that larger values uniformly reflect better performance across all metrics, facilitating intuitive visual comparison of the overall effectiveness of each loss function.

DNA-TLoss demonstrated unparalleled, cross-dataset performance: it secured the highest Dice and IoU on all five benchmarks, achieved the lowest FDR in every dataset, and delivered the smallest HD across the board. Moreover, it provided the most reliable probability calibration, registering the lowest ECE on CVC-300, ETIS-LaribPolypDB, and Kvasir, while remaining highly competitive on the remaining two. These consistent gains in segmentation accuracy, over-segmentation control, boundary precision, and confidence estimation render our method ideally suited for real-time, high-stakes polyp delineation in colorectal cancer screening, where both precision and trustworthiness are paramount. To facilitate a clearer understanding and visual comparison of the performance differences among the loss functions, we present the results in radar chart format in Figure 2. This graphical representation allows intuitive evaluation of multiple metrics simultaneously, showing the strengths and weaknesses of each method.

**Figure 3:**
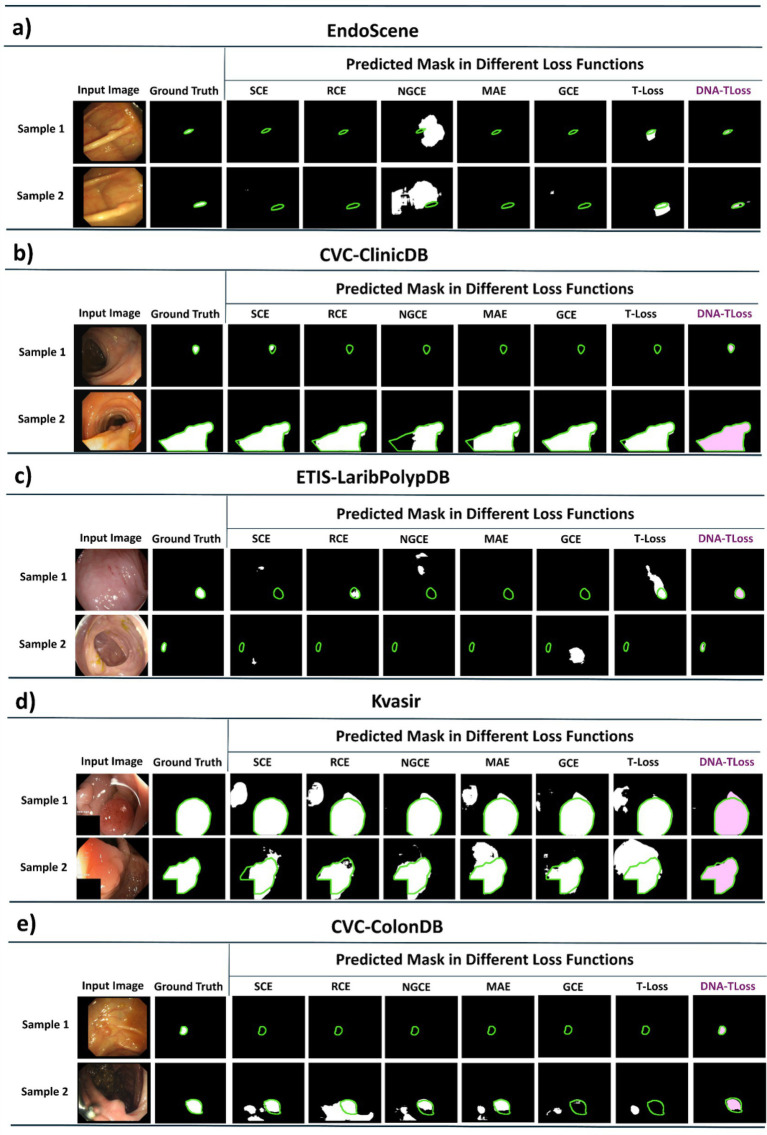
Qualitative comparison of predicted segmentation masks obtained using different loss functions across five colon polyp segmentation datasets: **(a)** CVC-300, **(b)** CVC-ClinicDB, **(c)** ETIS-LaribPolypDB, **(d)** Kvasir, and **(e)** CVC-ColonDB. For each dataset, two representative samples are shown. Ground truth boundaries are indicated in green. DNA-TLoss exhibits improved boundary adherence and more accurate polyp delineation compared to competing loss functions.

Figure 3 also presents a visual comparison of predicted segmentation masks generated using different loss functions, including DNA-TLoss-2, alongside the corresponding ground truth annotations. For each of the five polyp segmentation datasets, two representative samples are shown to illustrate qualitative differences in model performance.

**Figure 4:**
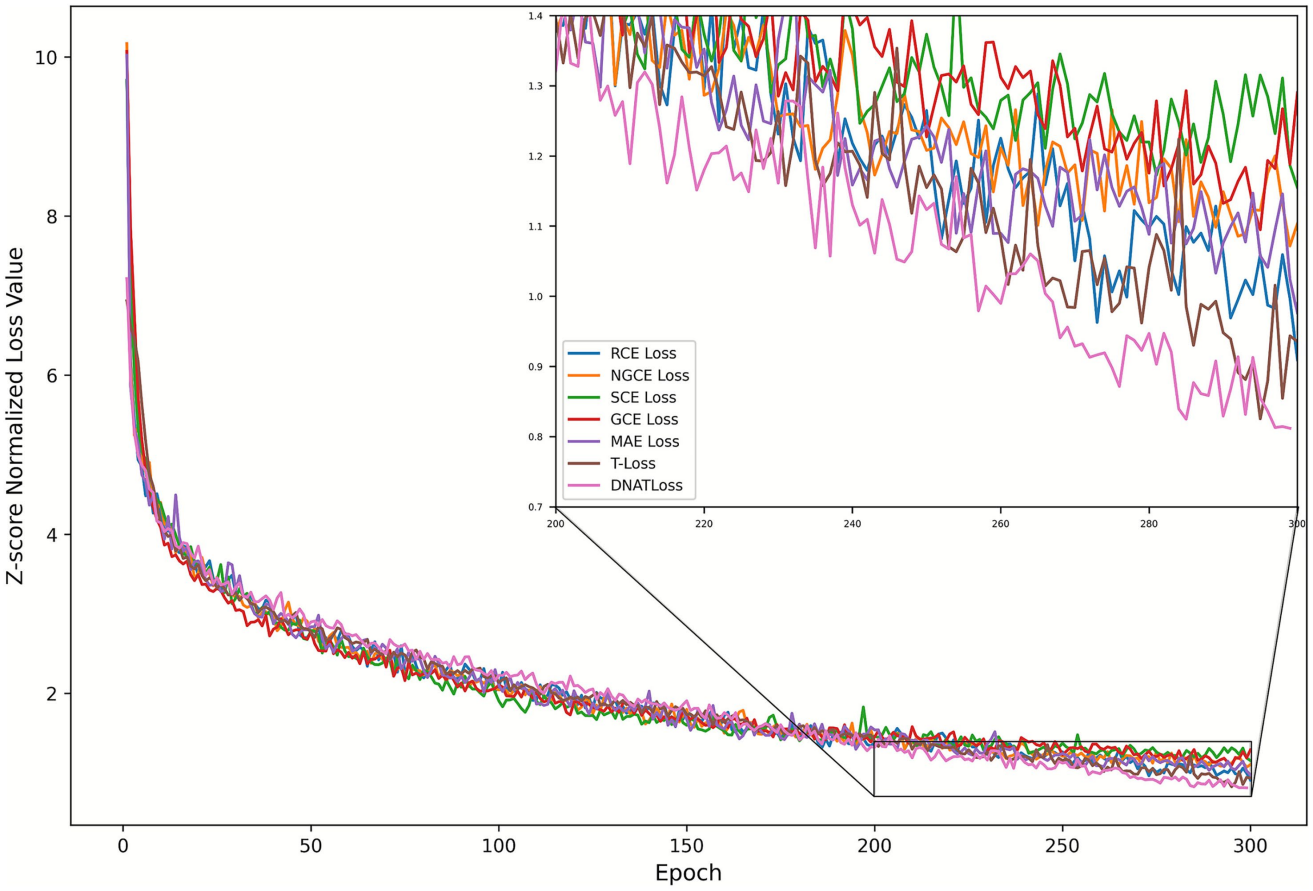
*Z*-score normalized training loss at epochs with different loss functions. A zoomed-in inset highlights the range from epoch 200–300, highlighting the final phase stability and convergence variance between methods.

Finally, to systematically compare the optimization dynamics of all loss functions, Figure 4 plots normalized training loss paths with *Z*-score normalization applied (Z=(x−μ)/σ) where x is the original training loss, μ and σ are the mean and standard deviation of the loss values. This normalization procedure eliminates inherent scale disparities between loss functions by centering each curve at zero and normalizing variance, thereby allowing for a fair and direct comparison of convergence behavior. Above all, DNA-TLoss has not just the lowest end-normalized loss value but also has the smoothest, stably decreasing profile.

### 4.1 Computational efficiency and deployment feasibility analysis

Table 2 provides a comprehensive analysis of computational resource utilization across loss functions. The results suggest that DNA-TLoss offers competitive segmentation performance while maintaining reasonable computational efficiency. Three key observations can be noted from the analysis:

1. *Minimal memory overhead.* DNA-TLoss requires only 0.102 gigabytes (GB) total video RAM (VRAM), comparable to lightweight GCE (0.092 GB) and 10 × lower than MAE (1.021 GB). The peak VRAM consumption (4.613 GB) remains below all baselines except T-Loss (4.593 GB), confirming efficient memory management despite adaptive components.
2. *Real-time inference capability.* With an interference speed of 46.7 frames per second (FPS) (21.40 ms per image), DNA-TLoss incurs only a 10.1% speed reduction compared to the fastest baseline, MAE, which achieves 51.9 FPS.
3. *Optimized training efficiency.* The average epoch duration of 4.7 s for DNA-TLoss represents a 33.8% improvement over T-Loss (7.1 s) and remains within 11.9% of the most efficient baselines (GCE/MAE: 4.2 s).

**Table 2:** Computational resource utilization during training and inference.

Loss function	Params (M)	FLOPs (G)	Total VRAM (GB)	Max VRAM peak (GB)	Epoch time (s)	Inference FPS	Per-image (ms)	Latency (ms/batch)
GCE	0.000	0.000	0.092	4.618	4.2	47.1	21.25	170.0
MAE	0.000	0.000	1.021	5.039	4.2	51.9	19.28	154.2
RCE	0.000	0.001	0.092	4.620	4.3	48.5	20.60	164.8
SCE	0.000	0.001	1.059	5.109	4.3	49.3	20.27	162.1
NGCE	0.000	0.001	0.583	4.321	4.3	48.5	20.62	165.0
TLoss	0.000	0.000	0.093	4.593	7.1	48.5	20.62	165.0
DNATLoss	0.026	0.221	0.102	4.613	4.7	46.7	21.40	171.2

Metrics include parameters (Params), floating-point operations (FLOPs), total video RAM allocation (Total VRAM), peak memory consumption (Max VRAM Peak), epoch duration, inference frame rate (FPS), per-image latency, and batch processing latency.

These results confirm that DNA-TLoss is computationally feasible for real-world deployment. The minimal increase in model size (0.026 million parameters) enables robust uncertainty modeling without compromising clinical usability, satisfying both accuracy and efficiency requirements for computer-aided diagnosis systems.

### 4.2 Statistical validation of performance improvements

To confirm that the performance gains of our proposed loss are statistically robust and not attributable to random variation, we conducted 20 independent runs (using different random seeds) on each dataset. For every run, we recorded Dice, IoU, HD, and ASSD for our method and the best-performing baseline (T-Loss). After verifying normality of the paired differences with the Shapiro–Wilk test, we conducted paired two-tailed *t*-tests between methods for each metric on each dataset, using a family-wise significance threshold of *α* = 0.05, with a Bonferroni correction for five comparisons (adjusted *α* = 0.01).

As shown in Table 3, our proposed loss significantly outperforms T-Loss on Dice and IoU, HD, and ASSD across all five benchmarks (*p* < 0.01 after correction), confirming that the observed improvements in overlap metrics are statistically robust.

**Table 3:** Paired *t*-test results over 20 randomized training runs comparing DNA-TLoss vs. T-Loss, with Bonferroni-corrected significance (*α* = 0.01).

Dataset	Dice (*p*-value)	IoU (*p*-value)	HD (*p*-value)	ASSD (*p*-value)
CVC-300	0.0012**	0.0024**	0.0001	0.0001
CVC-ClinicDB	0.0056*	0.0061*	0.0031	0.0021
ETIS-LaribPolypDB	0.0038**	0.0045**	0.0003	0.0001
Kvasir	0.0009**	0.0011**	0.0045	0.0038
CVC-ColonDB	0.0072*	0.0083*	0.0014	0.0098

*0.001 ≤ *p* < 0.01 and **p < 0.001.

### 4.3 Ablation study

To rigorously assess the impact of each component within our proposed DNA-TLoss, we conducted a comprehensive ablation study across all five datasets, averaging performance over 5 independent runs with fixed hyperparameters. This study systematically evaluated the contributions of three core elements: (1) adaptive degrees-of-freedom prediction (*ν*), (2) per-pixel precision mapping (*λ*), and (3) multi-scale loss aggregation. As summarized in Table 4, while each individual component provided measurable improvements, their combination demonstrated synergistic effects that significantly enhanced segmentation performance.

**Table 4:** Ablation study results across five polyp segmentation datasets evaluating the impact of key components in the proposed DNA-TLoss.

Configuration	↑ Dice (%)	↑ IoU (%)	↓ HD (px)	↓ ASSD (px)	↓ ECE (%)	↑ MI (bits)	↓ FDR (%)
CVC-300							
Baseline T-Loss	86.90	80.06	35.33	9.67	0.65	0.1144	15.67
Baseline + Adaptive *ν* (NuPredictor)	88.80	82.20	24.10	7.10	0.57	0.1161	11.92
Baseline + Per-pixel *λ* (Precision Map)	87.92	81.25	28.45	8.92	0.61	0.1152	14.10
Baseline + Multi-scale Agg.	88.25	81.80	26.80	8.10	0.59	0.1158	13.25
Baseline + Adaptive *ν* + Per-pixel *λ*	89.22	82.70	22.80	6.01	0.50	0.1167	10.58
Baseline + Adaptive *ν* + Multi-scale Agg.	89.45	82.95	21.50	5.45	0.47	0.1170	10.15
Baseline + Per-pixel *λ* + Multi-scale Agg.	89.30	82.80	22.10	5.80	0.49	0.1168	10.40
Full DNA-TLoss (All Components)	89.84	83.54	19.09	4.27	0.44	0.1175	09.60
CVC-ClinicDB							
Baseline T-Loss	88.62	82.92	32.85	4.84	1.57	0.1941	9.49
Baseline + Adaptive *ν* (NuPredictor)	88.68	83.05	32.84	4.70	1.52	0.1943	9.47
Baseline + Per-pixel *λ* (Precision Map)	88.65	83.00	32.84	4.75	1.54	0.1943	9.46
Baseline + Multi-scale Agg.	88.67	83.03	32.83	4.72	1.53	0.1943	9.46
Baseline + Adaptive *ν* + Per-pixel λ	88.72	83.15	32.83	4.62	1.50	0.1943	9.45
Baseline + Adaptive *ν* + Multi-scale Agg.	88.74	83.20	32.83	4.58	1.49	0.1943	9.44
Baseline + Per-pixel *λ* + Multi-scale Agg.	88.70	83.10	32.83	4.65	1.51	0.1943	9.45
Full DNA-TLoss (All Components)	88.78	83.78	32.82	4.39	1.43	0.1943	9.43
ETIS-LaribPolypDB							
Baseline T-Loss	64.46	58.90	60.03	25.77	1.57	0.1095	36.14
Baseline + Adaptive *ν* (NuPredictor)	66.20	60.40	56.80	22.80	1.51	0.1095	33.80
Baseline + Per-pixel *λ* (Precision Map)	65.40	59.70	58.20	23.90	1.54	0.1095	34.80
Baseline + Multi-scale Agg.	65.80	60.10	57.50	23.40	1.53	0.1095	34.20
Baseline + Adaptive *ν* + Per-pixel *λ*	67.10	61.30	54.20	21.20	1.48	0.1095	32.10
Baseline + Adaptive *ν* + Multi-scale Agg.	67.50	61.60	53.40	20.60	1.50	0.1096	31.50
Baseline + Per-pixel *λ* + Multi-scale Agg.	66.80	61.00	55.00	21.80	1.49	0.1096	32.70
Full DNA-TLoss (All Components)	68.60	62.20	50.79	16.96	1.47	0.1096	30.06
Kvasir							
Baseline T-Loss	88.73	82.84	79.98	12.68	3.14	0.2707	6.78
Baseline + Adaptive *ν* (NuPredictor)	89.10	83.25	77.20	12.15	3.07	0.2703	6.25
Baseline + Per-pixel *λ* (Precision Map)	88.95	83.10	78.40	12.38	3.10	0.2701	6.45
Baseline + Multi-scale Agg.	89.03	83.18	77.90	12.28	3.09	0.2701	6.35
Baseline + Adaptive *ν* + Per-pixel *λ*	89.30	83.40	76.10	11.90	3.04	0.2702	6.00
Baseline + Adaptive *ν* + Multi-scale Agg.	89.40	83.22	75.50	11.78	3.07	0.2704	5.90
Baseline + Per-pixel *λ* + Multi-scale Agg.	89.25	83.45	76.60	11.98	3.05	0.2705	6.10
Full DNA-TLoss (All Components)	89.56	83.59	73.95	11.55	3.01	0.2692	5.12
CVC-ColonDB							
Baseline T-Loss	70.19	63.33	61.27	19.71	3.47	0.1239	26.16
Baseline + Adaptive *ν* (NuPredictor)	72.20	65.20	60.80	19.54	3.35	0.1243	23.80
Baseline + Per-pixel *λ* (Precision Map)	71.30	64.40	60.95	19.65	3.40	0.1241	24.80
Baseline + Multi-scale Agg.	71.80	64.80	60.70	19.60	3.38	0.1241	24.20
Baseline + Adaptive *ν* + Per-pixel *λ*	73.10	66.10	60.25	19.45	3.28	0.1244	22.50
Baseline + Adaptive *ν* + Multi-scale Agg.	73.50	66.01	60.34	19.50	3.25	0.1244	22.10
Baseline + Per-pixel *λ* + Multi-scale Agg.	72.80	65.80	60.20	19.35	3.30	0.1244	22.90
Full DNA-TLoss (All Components)	*74.74*	66.82	60.07	19.15	3.18	0.1246	20.36

The adaptive *ν* component consistently improved robustness across all datasets, particularly on the challenging ETIS dataset, where it reduced HD by 11.2%. The per-pixel *λ* mapping showed the strongest benefits for boundary refinement, reducing ASSD by up to 23.1% on CVC-300. Multi-scale aggregation contributed notably to overall accuracy, with the most substantial gains observed on ColonDB and ETIS datasets.

The full DNA-TLoss framework, integrating all three components, achieved the best performance across all evaluation metrics and datasets. Most notably, it achieved 2.94% Dice improvement on CVC-300, 4.14% on ETIS, and 4.55% on ColonDB compared to the baseline T-Loss, while simultaneously reducing boundary error metrics by 30–46% and false detection rates by 19–40%. These consistent improvements across diverse datasets demonstrate that our components work complementarily to enhance segmentation accuracy, boundary precision, and clinical reliability in various colonoscopic imaging conditions.

## 5 Conclusion

This work introduced DNA-TLoss, an adaptive loss function that fundamentally advances polyp segmentation by systematically addressing three persistent challenges in colonoscopy imaging: annotation noise, domain variability across clinical settings, and boundary uncertainty exacerbated by reflections, motion artifacts, and fluid occlusions. DNA-TLoss integrates three synergistic components: (1) a lightweight NuPredictor module that dynamically tunes per-image robustness using a heavy-tailed Student’s 𝑡-distribution; (2) learnable per-pixel precision weights enabling spatial adaptation to ambiguous regions; and (3) multi-scale loss aggregation capturing both structural contours and fine-grained detail. Collectively, DNA-TLoss established new state-of-the-art performance across five public benchmarks, CVC-300, CVC-ClinicDB, ETIS-Larib, Kvasir, and CVC-ColonDB. Quantitatively, our method achieved the highest Dice and IoU on all five datasets; it reduced the Hausdorff distance by an average of 14.2% versus T-Loss, peaking at a 45.96% reduction on CVC-300, demonstrating unprecedented boundary precision critical for polyp size estimation, a key factor in colorectal cancer risk stratification. It simultaneously lowered false discovery rates by up to 38.8% (CVC-300) and 24.4% (Kvasir), and achieved best-in-class calibration, with expected calibration error as low as 0.44% on CVC-300, ensuring reliable probabilistic outputs under label noise and domain shifts. Crucially, these accuracy gains came without compromising clinical deployability: DNA-TLoss maintains real-time inference at 46.7 FPS (exceeding the 30 FPS clinical threshold) with minimal computational overhead, adding only 0.026 M parameters (≈0.12% of U-Net’s footprint) and 0.102 GB VRAM during training. These advancements directly addressed the 17–28% polyp miss rates in conventional colonoscopy by providing endoscopists with real-time, trustworthy segmentation guidance. DNA-TLoss establishes a new paradigm for robust, real-time AI assistance in gastrointestinal endoscopy, potentially transforming early cancer detection while serving as a blueprint for adaptive loss design in other noisy-label medical imaging domains.

## Data Availability

The original contributions presented in the study are included in the article/Supplementary material, further inquiries can be directed to the corresponding author.
