# Supplementary material for: Robust colonoscopy polyp segmentation using dynamic-Nu T-Loss with multi-scale and uncertainty-aware adaptation
Source: Front Med (Lausanne). 2026 Jan 12;12:1657123. doi: 10.3389/fmed.2025.1657123 (PMC12832355; doi:10.3389/fmed.2025.1657123)
Supplement: Supplementary file 1 [file Data_Sheet_1.zip › exported_notebook.html]

Notebook


In [ ]:

```
!pip install segmentation_models_pytorch
!pip install -U albumentations
```

In [ ]:

```
import math
import os
import cv2
import numpy as np
import torch
import torch.nn as nn
import torch.nn.functional as F
import torch.optim as optim
from torch.utils.data import DataLoader, Dataset
from torch.optim.lr_scheduler import LambdaLR
import segmentation_models_pytorch as smp
from tqdm import tqdm
import albumentations as A
from albumentations.pytorch import ToTensorV2
import argparse
import sys

# === Config ===
image_height = 512
image_width = 608
image_mean = np.array([0.557, 0.322, 0.236])
image_std = np.array([0.307, 0.215, 0.178])
num_workers = 4

class FeatureExtractor(nn.Module):
    """Lightweight CNN to extract features for nu prediction"""
    def __init__(self, in_channels=3):
        super().__init__()
        self.conv_layers = nn.Sequential(
            nn.Conv2d(in_channels, 16, 3, stride=2, padding=1),
            nn.ReLU(),
            nn.Conv2d(16, 32, 3, stride=2, padding=1),
            nn.ReLU(),
            nn.Conv2d(32, 64, 3, stride=2, padding=1),
            nn.ReLU()
        )
        self.pool = nn.AdaptiveAvgPool2d(1)
        
    def forward(self, x):
        features = self.conv_layers(x)
        return self.pool(features).squeeze(-1).squeeze(-1)

class DNATLoss(nn.Module):
    """Multi-Scale T-Loss with Dynamic Nu Adaptation"""
    def __init__(self, scales, weights, device, num_channels=1, 
                 nu_init=1.0, reduction="mean", epsilon=1e-8):
        super().__init__()
        self.scales = scales
        self.weights = weights
        self.device = device
        self.num_channels = num_channels
        self.reduction = reduction
        self.epsilon = epsilon
        
        # Feature extractor and nu predictor
        self.feature_extractor = FeatureExtractor()
        self.nu_predictor = nn.Sequential(
            nn.Linear(64, 32),
            nn.ReLU(),
            nn.Linear(32, 1),
            nn.Softplus()  # Ensure positive output
        )
        
        # Create lambda parameters for each scale
        self.lambdas_dict = nn.ParameterDict()
        
        # Move to device immediately
        self.to(device)
        
    def forward(self, input_tensor, target_tensor, original_image):
        """
        Compute multi-scale T-Loss with dynamic nu
        
        Args:
            input_tensor (torch.Tensor): Predicted probability map (B, C, H, W)
            target_tensor (torch.Tensor): Ground truth mask (B, 1, H, W)
            original_image (torch.Tensor): Original input image (B, 3, H, W)
        """
        # Extract features and predict nu
        features = self.feature_extractor(original_image)
        nu_pred = self.nu_predictor(features).squeeze()  # [B]
        
        total_loss = 0.0
        B, C, H, W = input_tensor.shape
        
        for weight, scale in zip(self.weights, self.scales):
            # Calculate resolution for this scale
            h = max(1, int(H * scale))
            w = max(1, int(W * scale))
            key = f"{h}_{w}"
            
            # Create lambda parameter if not exists
            if key not in self.lambdas_dict:
                self.lambdas_dict[key] = nn.Parameter(
                    torch.zeros(h, w, device=self.device)
                )
            
            # Get lambda for this resolution
            lambdas = self.lambdas_dict[key]
            
            # Resize input and target
            pred_resized = F.interpolate(input_tensor, size=(h, w), mode='bilinear', align_corners=False)
            target_resized = F.interpolate(target_tensor.float(), size=(h, w), mode='nearest')
            
            # Compute T-Loss for this scale
            loss_val = self.t_loss_function(
                pred_resized, 
                target_resized, 
                lambdas, 
                nu_pred,
                self.epsilon,
                self.reduction
            )
            
            total_loss += weight * loss_val
            
        return total_loss

    def t_loss_function(self, pred, target, lambdas, nu_pred, epsilon, reduction):
        delta_i = pred - target
        nu_positive = nu_pred + epsilon  # Use predicted nu
        B, C, H, W = pred.shape
        D = H * W * C

        # Calculate terms of T-Loss distribution
        first_term = -torch.lgamma((nu_positive + D) / 2)
        second_term = torch.lgamma(nu_positive / 2)
        third_term = -0.5 * torch.sum(lambdas + epsilon)
        fourth_term = (D / 2) * torch.log(torch.tensor(math.pi, device=pred.device))
        fifth_term = (D / 2) * nu_positive

        # Compute weighted squared differences
        delta_squared = torch.pow(delta_i, 2)
        lambdas_exp = torch.exp(lambdas + epsilon)
        lambdas_expanded = lambdas_exp.view(1, 1, H, W)
        numerator = torch.sum(delta_squared * lambdas_expanded, dim=(1, 2, 3))

        # Final terms
        fraction = numerator / nu_positive
        sixth_term = ((nu_positive + D) / 2) * torch.log(1 + fraction + epsilon)

        # Combine all terms
        total_losses = (
            first_term + 
            second_term + 
            third_term + 
            fourth_term + 
            fifth_term + 
            sixth_term
        )

        # Apply reduction
        if reduction == "mean":
            return total_losses.mean()
        elif reduction == "sum":
            return total_losses.sum()
        else:
            return total_losses
            
    # Add this method to get parameters
    def parameters(self):
        params = list(self.feature_extractor.parameters()) + list(self.nu_predictor.parameters())
        for key in self.lambdas_dict:
            params.append(self.lambdas_dict[key])
        return iter(params)

# === Dice Metric ===
def dice_score(preds: torch.Tensor, targets: torch.Tensor, threshold=0.5, epsilon=1e-6) -> float:
    """
    Computes Dice coefficient for binary segmentation.
    """
    # Convert to probabilities if needed
    if preds.shape[1] == 1:
        preds_probs = torch.sigmoid(preds)
    else:
        preds_probs = torch.softmax(preds, dim=1)[:, 1:2]  # For multi-class, take class 1

    # Binarize predictions
    preds_bin = (preds_probs > threshold).float()
    
    # Ensure targets have the same shape as predictions
    if targets.dim() == 3:
        targets = targets.unsqueeze(1)  # Add channel dimension if missing
    
    # Calculate Dice
    intersection = (preds_bin * targets).sum(dim=(1, 2, 3))
    union = preds_bin.sum(dim=(1, 2, 3)) + targets.sum(dim=(1, 2, 3))
    dice = (2. * intersection + epsilon) / (union + epsilon)
    return dice.mean().item()

# === Augmentations ===
def get_train_transforms():
    return A.Compose([
        A.Resize(image_height, image_width),
        A.HorizontalFlip(p=0.5),
        A.VerticalFlip(p=0.5),
        A.ElasticTransform(alpha=1.0, sigma=50.0, p=0.5),
        A.GridDistortion(num_steps=5, distort_limit=0.3, p=0.5),
        A.RandomBrightnessContrast(p=0.5),
        A.GaussNoise(p=0.5),
        A.Normalize(mean=image_mean, std=image_std),
        ToTensorV2()
    ])

def get_val_transforms():
    return A.Compose([
        A.Resize(image_height, image_width),
        A.Normalize(mean=image_mean, std=image_std),
        ToTensorV2()
    ])

# === Dataset ===
class MedicalSegmentationDataset(Dataset):
    def __init__(self, list_file, transforms=None):
        with open(list_file, 'r') as f:
            self.samples = [line.strip().split('\t') for line in f]
        self.transforms = transforms

    def __len__(self):
        return len(self.samples)

    def __getitem__(self, idx):
        img_path, mask_path = self.samples[idx]
        img = cv2.cvtColor(cv2.imread(img_path), cv2.COLOR_BGR2RGB)
        mask = cv2.imread(mask_path, cv2.IMREAD_GRAYSCALE)
        mask = (mask > 127).astype(np.float32)
        
        if self.transforms:
            sample = self.transforms(image=img, mask=mask)
            img, mask = sample['image'], sample['mask']
        else:
            img = torch.from_numpy(img).permute(2, 0, 1).float() / 255.0
            mask = torch.from_numpy(mask).float()
            
        return img, mask

# === Utility ===
def prepare_list(image_dir, mask_dir, output_file):
    image_files = sorted([f for f in os.listdir(image_dir) if f.lower().endswith(('.png', '.jpg', '.jpeg'))])
    mask_files = sorted([f for f in os.listdir(mask_dir) if f.lower().endswith(('.png', '.jpg', '.jpeg'))])
    assert len(image_files) == len(mask_files), "Mismatch in image and mask counts"
    with open(output_file, 'w') as f:
        for im, mk in zip(image_files, mask_files):
            f.write(f"{os.path.join(image_dir, im)}\t{os.path.join(mask_dir, mk)}\n")

# === Train/Val Loops ===
def train_epoch(model, loader, loss_fn, optimizer, device, loss_type='bce'):
    model.train()
    running_loss = 0.0
    for x, y in tqdm(loader, desc="Train"):
        x, y = x.to(device), y.to(device)

        # Ensure mask has channel dimension
        if y.dim() == 3:
            y = y.unsqueeze(1)  # [B, H, W] -> [B, 1, H, W]

        optimizer.zero_grad()
        out = model(x)  # [B, C, H, W]

        if loss_type == 'dna_tloss':
            # Convert to probabilities with channel dimension
            if out.shape[1] == 1:
                probs = torch.sigmoid(out)  # [B, 1, H, W]
            else:
                probs = torch.softmax(out, dim=1)[:, 1:2]  # [B, 1, H, W]
            # Pass original image to loss function
            loss = loss_fn(probs, y, x)
        elif loss_type == 'tloss':
            # Convert to probabilities with channel dimension
            if out.shape[1] == 1:
                probs = torch.sigmoid(out)  # [B, 1, H, W]
            else:
                probs = torch.softmax(out, dim=1)[:, 1:2]  # [B, 1, H, W]
            loss = loss_fn(probs, y)
        else:
            loss = loss_fn(out, y)

        loss.backward()
        optimizer.step()
        running_loss += loss.item()

    return running_loss / len(loader)

def val_epoch(model, loader, loss_fn, device, loss_type='bce'):
    model.eval()
    running_loss = 0.0
    running_dice = 0.0
    with torch.no_grad():
        for x, y in tqdm(loader, desc="Val"):
            x, y = x.to(device), y.to(device)
            
            # Ensure mask has channel dimension
            if y.dim() == 3:
                y = y.unsqueeze(1)  # [B, H, W] -> [B, 1, H, W]
            
            out = model(x)  # [B, C, H, W]
            
            if loss_type == 'dna_tloss':
                # Convert to probabilities with channel dimension
                if out.shape[1] == 1:
                    probs = torch.sigmoid(out)  # [B, 1, H, W]
                else:
                    probs = torch.softmax(out, dim=1)[:, 1:2]  # [B, 1, H, W]
                # Pass original image to loss function
                loss = loss_fn(probs, y, x)
            elif loss_type == 'tloss':
                # Convert to probabilities with channel dimension
                if out.shape[1] == 1:
                    probs = torch.sigmoid(out)  # [B, 1, H, W]
                else:
                    probs = torch.softmax(out, dim=1)[:, 1:2]  # [B, 1, H, W]
                loss = loss_fn(probs, y)
            else:
                loss = loss_fn(out, y)
                
            # Dice score uses original output shape
            dice = dice_score(out, y)
            running_loss += loss.item()
            running_dice += dice
            
    return running_loss / len(loader), running_dice / len(loader)
    
def main(args):
    device = torch.device('cuda' if torch.cuda.is_available() else 'cpu')

    if not os.path.isfile(args.train_list):
        prepare_list(args.train_img_dir, args.train_mask_dir, args.train_list)
    if not os.path.isfile(args.val_list):
        prepare_list(args.val_img_dir, args.val_mask_dir, args.val_list)

    # Always use raw logits for model output
    model = smp.Unet(
        encoder_name=args.encoder,
        encoder_weights='imagenet',
        in_channels=3,
        classes=2 if args.loss in ['tloss', 'dna_tloss'] else 1,
        activation=None
    )

    if torch.cuda.device_count() > 1:
        print(f"Using {torch.cuda.device_count()} GPUs")
        model = nn.DataParallel(model)
    model = model.to(device)

    # Define params based on loss type
    if args.loss == 'dna_tloss':
        scales = [1.0, 0.5, 0.25]
        weights = [1.0, 0.5, 0.3]
        loss_fn = DNATLoss(
            scales=scales,
            weights=weights,
            device=device,  # Pass device here
            num_channels=1,
            nu_init=args.nu,
            reduction="mean"
        )
        # Combine model parameters and TLoss parameters
        params = list(model.parameters()) + list(loss_fn.parameters())
    elif args.loss == 'tloss':
        scales = [1.0, 0.5, 0.25]
        weights = [1.0, 0.5, 0.3]
        loss_fn = TLoss(
            scales=scales,
            weights=weights,
            device=device,
            num_channels=1,
            nu_init=args.nu,
            reduction="mean"
        )
        # Combine model parameters and TLoss parameters
        params = list(model.parameters()) + list(loss_fn.parameters())
    else:
        loss_fn = nn.BCEWithLogitsLoss() if args.classes == 1 else nn.CrossEntropyLoss()
        params = model.parameters()

    train_ds = MedicalSegmentationDataset(args.train_list, transforms=get_train_transforms())
    val_ds = MedicalSegmentationDataset(args.val_list, transforms=get_val_transforms())
    train_loader = DataLoader(train_ds, batch_size=args.batch_size, shuffle=True, 
                              num_workers=num_workers, pin_memory=True)
    val_loader = DataLoader(val_ds, batch_size=args.batch_size, shuffle=False, 
                            num_workers=num_workers, pin_memory=True)

    optimizer = optim.Adam(params, lr=args.lr)
    total_iters = args.epochs * len(train_loader)
    scheduler = LambdaLR(optimizer, lr_lambda=lambda step: (1 - step / total_iters) ** args.poly_power)

    os.makedirs(args.save_dir, exist_ok=True)
    best_val = float('inf')

    for epoch in range(1, args.epochs + 1):
        train_loss = train_epoch(model, train_loader, loss_fn, optimizer, device, args.loss)
        val_loss, val_dice = val_epoch(model, val_loader, loss_fn, device, args.loss)
        scheduler.step()

        print(f"Epoch {epoch}: Train Loss {train_loss:.4f} | Val Loss {val_loss:.4f} | Val Dice {val_dice:.4f} | LR {scheduler.get_last_lr()[0]:.6f}")

        model_state = model.module.state_dict() if isinstance(model, nn.DataParallel) else model.state_dict()
        torch.save(model_state, os.path.join(args.save_dir, f"epoch{epoch}.pth"))
        if val_loss < best_val:
            best_val = val_loss
            torch.save(model_state, os.path.join(args.save_dir, "best.pth"))
            print(f"Saved new best model with val loss: {val_loss:.4f}")
```
